# Unknown Quantity: Regulating Radionuclides in Tap Water

**DOI:** 10.1289/ehp.120-a350

**Published:** 2012-08-31

**Authors:** Bob Weinhold

**Affiliations:** **Bob Weinhold**, MA, has covered environmental health issues for numerous outlets since 1996. He is a member of the Society of Environmental Journalists.


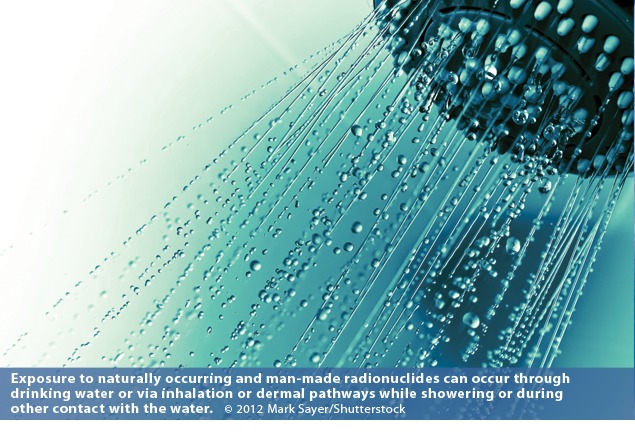
Radioactivity surrounds us; each day we are exposed to a certain amount by virtue of being alive on planet Earth. Some of this exposure comes from radioactive substances (radionuclides) that occur naturally in a wide variety of geologic and soil formations. Occasionally these naturally occurring substances become more concentrated or accessible through human activities such as mining and nuclear energy production, resulting in greater potential for exposure than their original natural occurrence would suggest. Other radionuclides are artificially created.

Residents in almost all parts of the United States live on lands that contain minor to substantial concentrations of radionuclides of one type or another.^^1^^ These substances often make their way into tap water, leading to exposures by ingestion, inhalation, or dermal pathways during showering or other contact with the water.

Although radionuclides are widespread, there are large gaps in our knowledge about sources of these materials, their distribution, associated health risks, and mitigation measures. However, the information we do have suggests that current drinking water standards for radionuclides established by the U.S. Environmental Protection Agency (EPA) may not adequately protect health. The EPA is set to review these standards relatively soon, and the next two years are prime time for filling in numerous information gaps and doing other legwork to make sure the review is as well informed as possible.

## Missing Science, Diverse Opinions

The EPA last revisited drinking water standards for radionuclides in 2000,^^2^^ when it established a maximum contaminant level (MCL) for uranium and reaffirmed requirements established in 1976^^3^^ for radium-226 and -228 combined, for gross alpha particle reactivity, and for beta particle/gamma ray radioactivity. By law, MCLs must balance information about health risks against the costs and limitations of available technologies. With the 2000 rule the EPA also established maximum contaminant level goals (MCLGs) of zero for radionuclides in drinking water. An MCLG is the concentration at which a chemical is believed to pose no adverse health risks.

The agency plans to complete a review of these standards by 2016 to determine if they need to be revised, expanded, or otherwise modified, says an EPA spokeswoman who asked to remain anonymous.^^4^^ Meanwhile, the EPA’s work on a radon standard for drinking water has been postponed since 1999,^^5^^ although in May 2012 the agency released a report to Congress laying out options for such a standard.^^6^^

The EPA has no plans to commission any particular studies prior to its next review of the radionuclide standards, according to its spokeswoman. As the agency works its way through the process of evaluating its standards—which the spokeswoman says now cover only the ingestion pathway, not inhalation or dermal routes—one starting point will be its current thresholds: 30 µg/L for uranium, 5 picocuries per liter (pCi/L) for combined radium-226 and -228, 15 pCi/L for gross alpha radioactivity, and 4 millirems per year for beta/gamma radioactivity.^^7^^

There is a wide range of opinion nationally and internationally for standards, guidelines, and health goals for substances such as uranium, radium, tritium, and alpha particles. Some of that diversity is reflected in the World Health Organization (WHO) guidelines for countries to use in regulating radionuclides in drinking water.^^8^^ That diversity is also reflected in actions some countries are taking to tighten their standards. For instance, in 2009 Canada adopted a uranium standard of 20 µg/L,^^9^^ and Germany in 2011 adopted an even lower standard of 10 µg/L.^^10^^

All radionuclides are a source of ionizing radiation and are known carcinogens.^^11^^^^,^^^^12^^ Zoltan Szabo, a research hydrologist with the U.S. Geological Survey (USGS), says the EPA threshold levels represent a reasonable guess as to levels associated with higher, perhaps less acceptable risks. “True, there may be effects not well characterized associated with even low detected levels, and those need further study,” he says. “But the observed catastrophic health effects have been associated with high levels of exposure.”

Szabo adds that zero exposure, although the stated goal of the EPA, is unlikely to ever be achievable and that reasonable threshold levels therefore need to be set. The California EPA, for instance, has calculated a Public Health Goal—comparable to a U.S. EPA MCLG—of 0.5 ppb (µg/L) for uranium in drinking water.^^13^^ This mathematically derived goal was estimated to provide *de minimis* risk against cancer as well as all noncancer effects.

Studies have focused on the carcinogenicity of radionuclides, as it is the phenomenon that has readily been identifiable in individuals exposed to large doses in the past. However, many other possibilities related to radionuclide exposure remain unexplored and possibly unaddressed by current U.S. and global standards, such as cumulative, long-term effects of multiple pathways and sources (including inhalation, ingestion, and dermal absorption from water, food, occupational exposures, background exposures, and accidents); interactions among multiple radionuclides and with other chemicals or microbes; effects on people with underlying health problems; and impacts on development and the endocrine, immune, and nervous systems.

But although major gaps in the science remain, studies and reviews since 2000 suggest the radiologic and chemical toxicity of radionuclides may be farther-reaching and more significant than thought at the time the EPA drinking water standards were established. For instance, the WHO and the U.S. National Research Council have concluded radionuclides follow a linear no-threshold model of carcinogenicity; that is, there is evidence that ionizing radiation can increase the risk of cancer at even the lowest doses.^^10^^^^,^^^^14^^

In framing adequately protective regulations, some experts believe greater attention needs to be extended to vulnerable populations such as children. “Our whole [regulatory] perspective is geared to cancer and adults,” says Arjun Makhijani, president of the Institute for Energy and Environmental Research, a Maryland-based advocacy group. “For instance, we’re not considering *in utero* doses at all.”

Others point to the need for more attention to noncancer effects in setting drinking water standards. Ellen Silbergeld, a professor of environmental health sciences and epidemiology at the Johns Hopkins Bloomberg School of Public Health, says there’s a critical shortage of information for all population groups on the chemical toxicity potential of most radionuclides. “I think this is a sleeper story,” she says. “A lot [of radionuclides] may possess considerable toxicity as metals. The research hasn’t really been done.”

## A Dirty Dozen

Among the radionuclides gaining the most research attention due to perceived health threat, frequency of occurrence, and other considerations are americium, cesium-137, cobalt-60, iodine-131/-129, lead-210, plutonium, polonium, radium, radon, strontium-90, tritium, and uranium (isotopes are listed except when all isotopes of an element are radioactive). All are metals except the gases radon and tritium and the halogen iodine. Of these, radon occurs only naturally, and americium, cesium-137, cobalt-60, plutonium-239, and strontium-90 are exclusively man-made. The remainder are both naturally occurring and man-made, although naturally occurring amounts of some of these elements may be infinitesimally small.^^15^^^^,^^^^16^^^^,^^^^17^^

Daniel Hirsch, president of the California-based advocacy group Committee to Bridge the Gap and a lecturer in nuclear policy at the University of California, Santa Cruz, points out that the distinction between naturally occurring and man-made radioactivity is more complicated than it may seem at first. For example, he says, if one finds radium-226 in drinking water, it is a naturally occurring radionuclide—that is, it is generally not created from some other element by human action—but the concentration of it may have been substantially enhanced by human activity. “Radium in water that got there just because there is a little bit in soil is one thing,” he says. “Radium in water that got there because it leaked from a tailings pile or contaminated radium processing facility is something else.”

Examples of human activities that may lead to radionuclide exposure include mining, milling, and processing of radioactive substances; wastewater releases from the hydraulic fracturing of oil and natural gas wells; and the manufacture, use, disposal, and/or accidental release of products such as nuclear fuel, nuclear weapons, military armor, phosphate fertilizer, and certain medical devices, smoke detectors, and plastics.

Many agencies have adopted the practice of measuring gross alpha and beta/gamma radioactivity in lieu of initially measuring for individual radionuclides. These measures reflect the presence of radionuclides that emit these particles or rays, such as polonium-210, cesium-137, and strontium-90.^^18^^ Each type of emission has different actions and potential health consequences. Alpha particles tend to be emitted by naturally occurring radionuclides, and beta particles and gamma photon rays tend to be emitted by man-made ones, although there are exceptions in each category.^^19^^^^,^^^^20^^

According to the EPA, most human exposures are to naturally occurring sources, including radon gas.^^7^^ Detectable concentrations of radionuclides are present in a high percentage of U.S. drinking water utility systems and individual wells, as well as water supplies in many other parts of the world.

In a limited study of radionuclides in wells used for drinking water, the USGS found radium levels above the current EPA standard in 3% of untreated water samples it assessed, including more than 20% of samples in certain areas. Among the states with wells that exceeded the standard were Arkansas, Florida, Georgia, Iowa, Massachusetts, New Hampshire, New Jersey, and Texas. About 20 other states had wells with detectable radium below the limit.^^21^^

In another USGS study, uranium was detected in about half the wells sampled and exceeded the EPA MCL in 4% of the wells. Among the states with wells exceeding the EPA standard were Arizona, California, Colorado, Florida, Kansas, Maine, Nebraska, Nevada, New Jersey, New Mexico, North Dakota, Texas, Utah, and Wyoming.^^22^^

In a third study focusing on the Northeast, the USGS found generally similar results, with some variations. Of all the domestic and public wells studied, exceedances of EPA standards occurred for uranium in 4–17% of wells sampled, for combined radium in 3–4% of wells sampled, for gross alpha radioactivity in 12–16% of wells sampled, and for beta radioactivity in 9% of wells sampled.^^23^^ Connecticut, Maine, Massachusetts, New Hampshire, New Jersey, Rhode Island, and Vermont each had a few wells with one or more exceedances.

The U.S. Centers for Disease Control and Prevention’s biomonitoring program^^24^^ has detected uranium in the urine of 92.5% of the population tested. This uranium would have come from all types of sources, not just tap water.

Jay Dempsey, a spokesman with the Agency for Toxic Substances and Disease Registry, says the urine levels are quite low and are not known to be associated with specific adverse health effects. “We all are exposed throughout life to low levels of radioactive materials in drinking water that do not appear to cause adverse health effects,” he explains. None of the dozen other radionuclides drawing substantial attention in the United States and around the globe are included in the biomonitoring.

**Figure:**
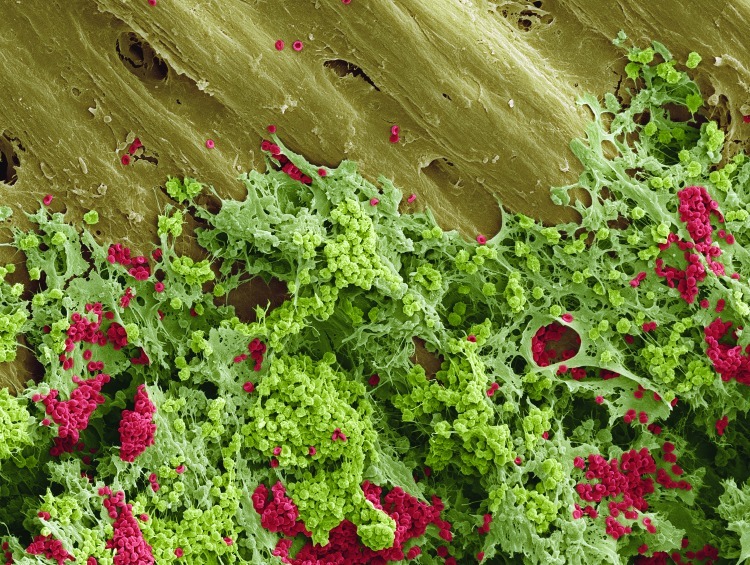
Scanning electron micrograph of bone marrow © 2012 Steve Gschmeissner / Photo Researchers, Inc.

## Consumer Uncertainty

Almost all water utilities are required to tell their customers, via an annual water quality report called the Consumer Confidence Report (CCR), about the substances detected in the water supply throughout the previous year.^^25^^ Management of this process varies somewhat by state; as a result, some utilities may deliver certain levels of radionuclides to their customers but report that fact only sporadically, or never.

It’s common for a utility to initially screen for radionuclides other than uranium and combined radium by testing for gross alpha and/or beta/gamma radioactivity. No additional testing is required unless detected radioactivity exceeds a certain threshold, which means regulators (and customers) do not know exactly which substances are causing those low levels of radioactivity. As the WHO explained in its guidance document, “The process of identifying individual radionuclides in drinking-water and determining their concentration is time-consuming and expensive. Because, in most circumstances, the concentrations are low, such detailed analysis is normally not justified for routine monitoring.”^^8^^ But Hirsch calls this “a pretty poor practice,” adding, “What you want to know is the particular radionuclide, since each has different toxicity.”

In other circumstances, utilities are required to test for specific substances only at intervals of five years or more, and detected substances must be reported only in the year for which they were tested. And when utilities do test, they typically are required to test only one moment per year, providing a fleeting snapshot of concentrations that can vary considerably over the course of a year. The EPA spokeswoman says the agency has no plans to change any of these aspects of CCR reporting.

Another challenge to accurate monitoring is that substances such as radium and sometimes strontium-90 can become attached to pipes in the system and be released as the chemistry of the water changes, says R. William Field, a professor of occupational and environmental health at the University of Iowa. Such releases are downstream from where the utility is required to test, so the spikes aren’t captured in the data. In addition, radium-adsorbed pipe scale presents an additional source for radon exposure that is not captured by point-of-release testing.^^26^^

When levels of concern are discovered, treatment costs can run into many millions of dollars for a utility, using methods such as reverse osmosis, ion exchange, coagulation, or lime softening.^^7^^ The treatment method selected depends on the extent of mitigation needed, the specific radionuclides targeted, the chemical characteristics of the water, the traits of the existing system, and other factors.

Consumers have their own options if they want their tap water to contain lower concentrations of radionuclides. These can range from installing a reverse osmosis filter at the kitchen faucet themselves, which would address the bulk of most people’s ingested water, to having a contractor install a whole-house reverse osmosis system. Depending on the number and type of filters, costs can range from a few hundred to tens of thousands of dollars.

As far as research needs go, a commentary in the December 2011 issue of *EHP* points to some of the work needed to gain a better understanding of health effects of radionuclides at exposures typically encountered in drinking water.^^27^^ In their overview of important gaps to fill and ways to do so, the commentary authors recommended better addressing possible confounding factors via thorough biochemical analyses of water being consumed and collection of data on other potential sources of radiation, such as diet, smoking, occupational exposures, and radiation from radon or other sources in the home.

The authors also said improvements are needed in methods for assessing duration, location, and effects of internal exposure, including identification of the most specific, sensitive, feasible biomarkers. And as with any other type of research, study populations (including sensitive subsets of people) need to be structured from the outset so they provide adequate statistical power. The authors note that to date, most studies in this area have suffered from limitations in exposure assessment that make it hard to tease out links between exposures and health effects.

**Of Leading Concern**

There are hundreds of radionuclides, but relatively few are regularly encountered. People typically are exposed to radionuclides that originate in sources such as tap water and food, from medical and occupational exposures, and occasionally from military, energy, waste, and similar sites. In the list below, where an element is listed rather than an individual radionuclide, the element has several radioactive isotopes of interest.

**Americium** is a by-product of plutonium production, and gets into the environment through nuclear weapons and energy production and use, as well as other manufacturing operations. It also is used in industrial moisture density gauges, certain smoke detectors, and medical diagnostic devices. It can lodge in bones, the liver, and other organs, and remain inside the body for decades.^28^

**Cesium-137** is the man-made radioactive form of this common metal, which acts like potassium in the body and can penetrate numerous cell types. It typically is eliminated in a matter of months. It may occur in the vicinity of nuclear weapons testing, accidents, mining, and milling.^29^

**Cobalt-60** occurs as a by-product of nuclear reactor operations and is a significant component of reactor waste. It also is used in several industrial and medical applications. It lodges largely in the bones, liver, and kidneys, and is considered quite potent in increasing cancer risk. The metal has also been linked with developmental problems and may contribute to adverse effects on the immune and cardiovascular systems. Children may be more vulnerable than adults.^30^

**Iodine-131** is largely man-made, while **iodine-129** can be either natural or man-made. Although radioactive iodine can cause health problems, it is also used in some medical applications. Most concern about toxic effects is focused on risk of thyroid damage or cancer. Children are considered more vulnerable than adults. Preventing or reducing uptake can be achieved through use of certain iodine compounds for short periods of time.^31^

**Lead-210** is a naturally occurring radionuclide that occurs in air, soils, rocks, and water. Its chemical toxicity is the same as that of stable lead, which is widely acknowledged as causing a range of harmful effects even at very low concentrations. The prevalence of lead-210 relative to stable isotopes of lead is quite low, and the risk posed as a carcinogen therefore is the main item of concern for this radionuclide.^32^

**Plutonium** occurs in infinitesimal amounts naturally, and is most likely to be found in drinking water due to human activities related to nuclear power and weapons, research facilities, waste disposal sites, and accidents. The fallout from atmospheric testing of nuclear weapons in the 1950s and ’60s dispersed plutonium around the world. It can migrate to the bones, liver, lungs, and elsewhere, where it can stay for decades. It has been linked with reduced immune function and kidney damage.^33^

**Polonium** is a highly radioactive carcinogen, but it hasn’t received widespread attention as a drinking water contaminant. However, polonium-210 has been found in drinking water in at least five widely dispersed U.S. states (Florida, Louisiana, Maryland, Nevada, and Virginia), sometimes at elevated concentrations in the context of the limited data. Polonium-210 occurs naturally or can be produced in a nuclear reactor. It can lodge in the liver, kidney, bone marrow, spleen, gastrointestinal tract, and gonads, and has been linked with adverse reproductive effects. Chronic intake can lead to increased absorption through the digestive tract, possibly due to intestinal wall damage caused by alpha particle emissions.^34^^,^^35^

**Radium** occurs widely in water and occasionally in air as a dust or aerosol. As with all radionuclides, it can be inhaled (via pathways such as shower mist), ingested, or absorbed through intact or damaged skin. It can stay in bones for many years and can lodge at a higher rate during periods of rapid growth, potentially affecting children more than adults. In addition to being a carcinogen and mutagen, it also has been linked with adverse reproductive, developmental, liver, kidney, and eye effects. Phosphate mining and fertilizer production can increase concentrations and exposures to radium and its daughter compound radon.^36^

**Radon** is a widespread, naturally occurring gas that can dissolve into water, sometimes at elevated concentrations, and is released into the air through showers and running faucets. In most parts of the United States this is considered a minor contributor to indoor radon concentrations, compared with other sources such as soils and rocks beneath a building, but it may be a concern in some settings. Inhaled radon is a leading cause of lung cancer, a risk exacerbated for those who smoke. Ingested radon can cause stomach cancer.^37^

**Strontium-90** has been emitted widely by nuclear power plants, weapons facilities, waste sites, and U.S. Department of Energy facilities. It has been linked with bone damage and adverse effects on the cardiovascular and immune systems. Children may be more vulnerable, as might those who have chronic kidney problems, diabetes, rheumatoid arthritis, or certain bone diseases, or who are on protein-deficient diets or drink alcohol heavily.^38^

**Tritium**, as a natural isotope of hydrogen, is ubiquitous. The vast majority of man-made tritium is generated by nuclear weapons and reactors, but it also is used in certain electronic components and emergency exit signs. It can enter various organs, fluids, and tissues within minutes, and can take several years to be fully eliminated from the body. It has been linked with adverse effects on bone marrow, reproduction, and development.^39^

**Uranium** is one radioactive element for which chemotoxic effects have been demonstrated; in fact, its effects on the kidneys are more severe than its radiotoxic effects, a risk reflected in the EPA drinking water standard promulgated in 2000. Uranium can be inhaled on dust particles or aerosols or ingested. It can also be absorbed through damaged or intact skin to varying degrees. Mining and hydraulic fracturing, or “fracking,” can concentrate levels of uranium (as well as radium, radon, and thorium) in wastewater.

In the body, uranium moves widely, and much of it leaves relatively quickly compared with many other radionuclides. The remainder ends up in locations such as the kidney, liver, and bones. Exposure has been linked with birth defects, certain types of genetic, developmental, and metabolic damage, and adverse effects on the kidneys, liver, and neurologic, endocrine, immune, reproductive, and cardiovascular systems.^40^

More research is needed to determine the extent to which these findings represent real-world human exposures. For instance, even with the breadth of information for uranium, the Agency for Toxic Substances and Disease Registry acknowledges there is little information on the full range of possible health effects, especially those resulting from oral and dermal exposures for humans. According to the Centers for Disease Control and Prevention, avoiding harmful concentrations is particularly important for uranium, because there are no known, effective ways to reduce body burden from chronic exposures.

**Unexplored Mechanisms**

Because many radionuclides lodge for long periods of time in bone, and because bone marrow plays a critical role in immune system function, there are concerns that these substances could cause immune system damage. One key immune system component in the bone marrow is pre-B cells, which are highly autoreactive.^41^

At least two studies have hinted that chemical or radiologic damage to these cells could contribute to autoimmune diseases. One found links between chronic or acute exposure to uranium and autoimmune thyroiditis or orchitis.^42^ Another found links between depleted uranium exposure and effects on immune cells and genes that can play a role in autoimmune diseases.^43^

Another fledgling line of inquiry involves possible interactions among toxics and infectious diseases. Initial forays into this niche suggest these interactions may be significant. For instance, Ellen Silbergeld of the Johns Hopkins Bloomberg School of Public Health and colleagues have observed that mercury has been linked with either positive or negative health effects in people when acting in concert with malaria or coxsackie B3 virus. Similar interactions have been seen for coal tar exposure and Shope papilloma virus (a rabbit pathogen), smoking and human papilloma-virus, and aflatoxin and hepatitis B virus.^44^

But the scant evidence in this field means it’s premature to speculate whether radionuclides might act similarly. “One should be careful about generalizing,” Silbergeld says. “It’s most important to prod people into doing more work.” However, she says, in her own studies of malaria and mercury exposure,^44^ “it’s been a very fruitful line of thought.”

Another biological realm possibly worth investigating more is the gut microbiome. There are indications of possible interactions among gut organisms and toxics such as arsenic, bisphenol A, polycyclic aromatic hydrocarbons, 2,2',4,4'-tetrabromodiphenyl, and some pharmaceuticals.^45^ Anthony Hay, an associate professor of microbiology at Cornell University, says he isn’t aware of any similar research on radioactive substances. However, he notes that at least one species of *Citrobacter*, a bacterium often found throughout the body, is known to accumulate uranium.^46^^,^^47^^,^^48^ But he says there is no way to know if this could lead to uranium toxicity in an infected person. “I only mention it as a proof of principle and do not want to imply that it is a likely candidate,” he says.

Major knowledge gaps in these potential toxicity pathways and mechanisms for radionuclides, and in the more commonly studied ones, contribute to the need for more research, wrote a team of French researchers in the December 2011 issue of *EHP*.^27^ Prospective studies that employ appropriate means of exposure monitoring and biochemical analysis of drinking water will be important for strengthening the body of research in this area, as will research to identify better biomarkers of radionuclide-induced health effects.

One tool that could conceivably help with such research is the biomonitoring program of the Centers for Disease Control and Prevention. However, the existing uranium data do not distinguish the different isotopes, says spokesman Jay Dempsey, making it impossible to identify the different radiologic effects of each. The same lack of isotope information holds true for cesium, cobalt, and lead, which are tracked collectively for all stable and radioactive isotopes. Dempsey says there are no plans to track any of these radioactive isotopes or to add any radionuclides.
